# Clinical outcomes and complications after anterior cruciate ligament reconstruction with bone–patellar tendon–bone in patient Tanner 3 and 4: a systematic review

**DOI:** 10.1007/s00590-022-03402-z

**Published:** 2022-10-29

**Authors:** Marco Turati, Marco Caliandro, Diego Gaddi, Massimiliano Piatti, Luca Rigamonti, Nicolò Zanchi, Paolo Di Benedetto, Linda Boerci, Marcello Catalano, Giovanni Zatti, Matthieu Ollivier, Marco Bigoni

**Affiliations:** 1 Orthopedic Department, San Gerardo Hospital, Monza, Italy; 2 School of Medicine and Surgery, University of Milano-Bicocca, Via Pergolesi 33, 20900 Monza, Italy; 3 Transalpine Center of Pediatric Sports Medicine and Surgery, University of Milano-Bicocca - Hospital Couple Enfant, Monza (Italy), Grenoble, France; 4 Department of Paediatric Orthopedic Surgery, Hospital Couple Enfants, Grenoble Alpes University, Grenoble, France; 5 Medical Department (DAME), University of Udine, Udine, Italy; 6 Clinic of Orthopaedics, Friuli Centrale Healthcare and University Trust (ASUFC), Udine, Italy; 7 Department of Orthopedic Surgery, Mayo Clinic, Rochester, MN USA; 8 Department of Orthopedics and Traumatology, St. Marguerite Hospital, APHM, CNRS, ISM, Institute of Movement and Locomotion, Aix Marseille University, Marseille, France; 9 Department of Orthopaedic and Trauma, Policlinico San Pietro Hospital, Ponte San Pietro, Bg, Italy; 10 Department of Orthopaedics & Rehabilitation, College of Medicine, The Pennsylvania State University, University Park, PA USA; 11 Department of Orthopedics and Traumatology, Clinica Ars Medica, Gravesano, Ticino, Switzerland

**Keywords:** Anterior cruciate ligament, Bone–patellar Tendon–bone graft, Growth plates, Adolescent, Arthroscopy

## Abstract

**Background:**

Clinical outcomes and potential complications associated with Bone–Patellar Tendon–Bone (BPTB) graft in skeletally immature ACL reconstruction (ACLR) are poorly defined. Considering that in Tanner 1–2 patients this kind of graft is not recommended, we focused our systematic review on the evaluation of all the studies in the literature that reported clinical outcomes and rate of complications of the ACLR using BPTB graft in Tanner 3–4 patients.

**Methods:**

This review was conducted in accordance with the PRISMA statement. PubMed, Cochrane Library, EMBASE and Scopus were examined from 1965 to 2020 using different combinations of the following keywords: “ACL reconstruction”, “skeletally immature”, “young”, “patellar tendon” and “BPTB”. The database search yielded 742 studies, on which we performed a primary evaluation. After carrying out a full-text evaluation for the inclusion criteria, 4 studies were included in the final review and assessed using the Newcastle–Ottawa scale. Ninety-six cases with mean age of 14.2 years were reported.

**Results:**

Good stability and functional outcomes were reported with a mean follow-up of 49.5 months. Return to sport rate ranged from 91.7% to 100%. A KT-1000 side-to-side difference higher than 5 mm was observed in five patients (5.2%). No lower limb length discrepancy and angulation were reported. Graft rupture rate was 5.2%.

**Conclusion:**

According to these results, BTPB graft could be a good choice in Tanner 3–4 patients who want to achieve their preinjury sport level with a low risk of growth disturbances and graft failure. Further investigations in a wider population are needed.

## Introduction

Anterior cruciate ligament (ACL) injury is common in children and adolescent and its incidence is getting higher year by year [1, 2]. In a population between 6 and 18 years old, the incidence is 121/100,000 per year, with slight differences between male (114/100,000) and female (129/100,000). ACL injury is also one of the most common injuries in paediatric population representing 6.7% of all injuries and 30.8% of all knee injuries in soccer players between 5 and 18 years old [3, 4]. Age is an important ACL tear risk factor with an average rate of incidence that increased by 2.3% every year, reaching peak in 16-year-old females and in 17-year-old males [5]. Sports that require cutting movements like football, soccer or basketball are at higher risk of ACL injury too with an incidence that varies between 1 and 3.4% [6].

ACL deficient knees are related to poor long-term outcomes including low objective IKDC scores, increased anterior tibial translation at arthrometry and radiological evaluation, increased joint laxity and extensive arthritic changes in the injured leg [7]. McCarroll et al. found that adolescents with ACL deficiency treated conservatively experienced recurrent instability, effusion and pain during activities [8]. Moreover, an ACL deficient knee often leads to secondary meniscal and/or cartilage damage, which may lead to knee degeneration and functional instability [9].

Different approaches are available in skeletally immature patients to restore ACL function and prevent potential damage of the growth plates [10, 11]. Physeal sparing surgical techniques are the most common among open physis patients including all-epiphyseal and extra-articular reconstruction [12], partial transphyseal and transphyseal reconstruction with soft tissue graft are mainly used in patients with low growth potential [13]. However, recent data suggest that drilling across open proximal tibial or distal femoral physes can be a safe and effective procedure in patients close to skeletal maturity. Animal studies show that risk of growth plate disturbance is related to tunnels of 7–9% of the cross-sectional area of the physis. Small and centrally placed tunnels are recommended to a minimizes the risk of physeal closure [14]. Moreover, transtibial techniques reduce the risk of femoral growth plate violations compared to anteromedial approach that could produce an elliptical tunnel with a larger and more lateral growth plate violation [15].

In immature patients, the main concern about growth plates is the risk of bone bridging and deformities. Considering that, some surgeons prefer soft tissue graft as hamstring for ACLR in skeletally immature patients [16–19]. An MR imaging study after transphyseal reconstruction of the ACL in skeletally immature adolescent patients shows a focal bone bridge in 11% of patients. However, no growth disturbances were observed in these patients [20].

Despite the large amount of research about ACL injury in skeletally immature patients, the choice regarding the best kind of graft for reconstruction is still widely debated [21]. Ligamentization process, long-term results and risk of re-rupture are not well defined yet. However, a high re-rupture risk after reconstruction with soft tissue graft in paediatric patients was reported in selected techniques [22–24]. Low re-rupture rate was reported in adult ACLR with BPTB, however clinical results, lower limb discrepancy rate and re-rupture rate of BPTB ACLR in young population are not well defined [25].

The aim of our systematic review is to evaluate all the studies in the literature that reported clinical outcomes and rate of complications of the ACLR using BPTB graft in almost skeletally mature patients.

## Materials and methods

### Focused question based

Based on the Preferred Reporting Items for Systematic Review and Meta-analysis (PRISMA) guidelines [26], we stated three specific questions: (1) What are the clinical outcomes of ACLR using BPTB in skeletally immature patients? (2) What is the reinjury rate after this technique? (3) How many complications are reported after ACLR using BPTB graft in skeletally immature patients?

### Eligibility criteria

**Fig. 1:**
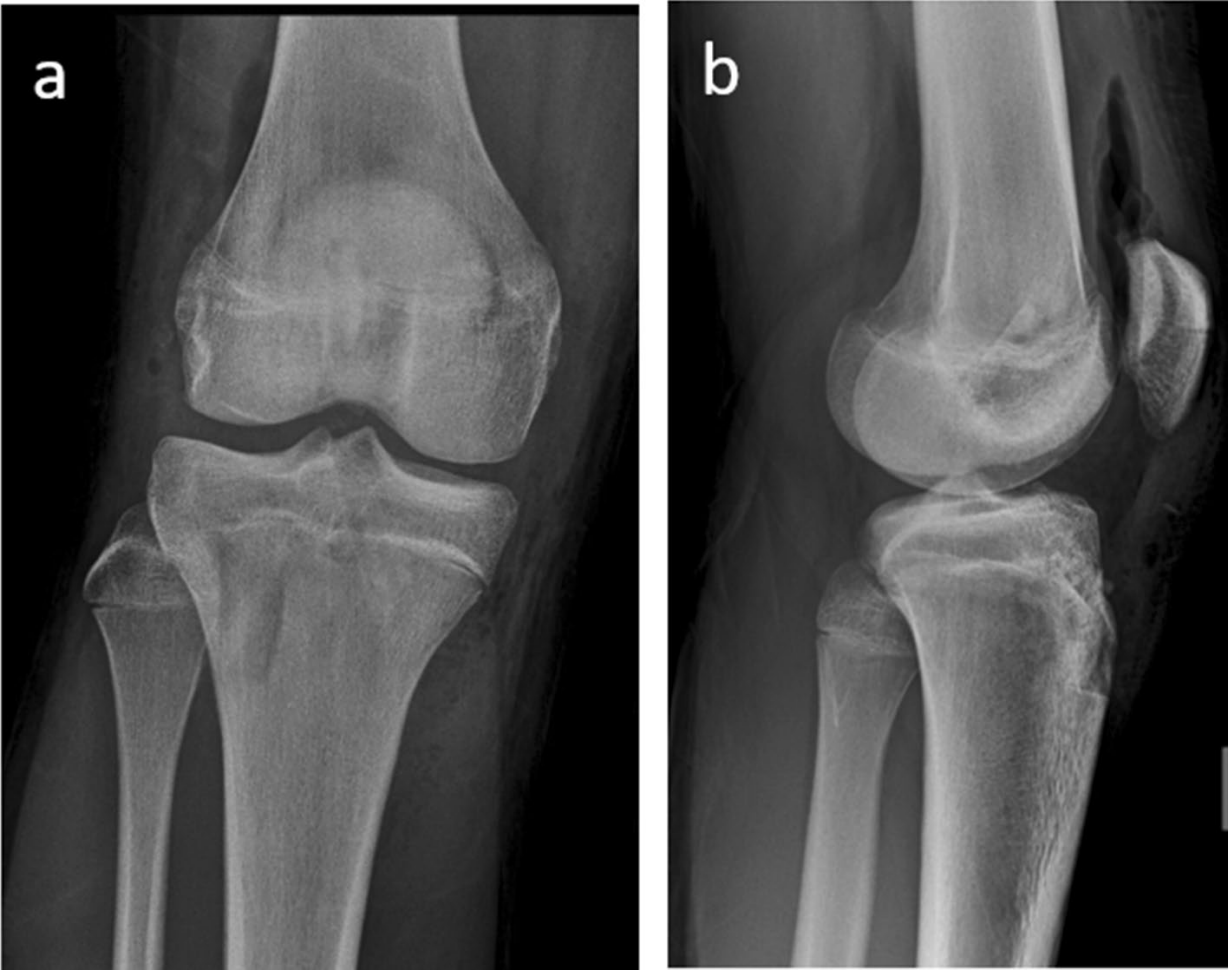
Postoperative X-rays of ACL reconstruction with BPTB in 15-year-old boy male. Anterior–posterior (**a**) and lateral (**b**) X-rays of the right knee show open physes and tunnels

The following inclusion criteria were used to determine study eligibility: (i) original clinical studies, (ii) case–control and cohort study, (iii) skeletally immature patients, (iv) patients with ACL injury and (v) type of intervention: transphyseal ACLR using patellar tendon (Fig. 1). Letters to the editor, historic reviews, case reports, case-series and unpublished articles were excluded.

### Search strategy

We analysed the literature from 1965 to May 2020 using a browser that search into several database including PubMed/Medline (National Library of Medicine, Washington, DC), Embase, Cochrane Library and Scopus, using the following combination of keywords: (a) “ACL injury” AND “skeletally immature” AND “patellar tendon”, (b) “ACL tear” AND “skeletally immature” AND “patellar tendon”, (c) “ACL injury” AND “skeletally immature” AND “BPTB”, (d) “ACL tear” AND “skeletally immature” AND “BPTB”, (e) “ACL injury” AND “young” AND “BPTB”, (f) “ACL tear” AND “young” AND “BPTB”, (g) “ACL injury” AND “young” “patellar tendon”, (h) “ACL tear” AND “young” AND “patellar tendon”, (i) “ACL reconstruction” AND “skeletally immature” AND “patellar tendon”, (l) “ACL reconstruction” AND “skeletally immature” AND “BPTB”, (m) “ACL reconstruction” AND “young” AND “BPTB”, (n) “ACL reconstruction” AND “children” AND “BPTB”, (o) “ACL reconstruction” AND “adolescent” AND “BPTB”, (p) “ACL injury” AND “children” AND “BPTB”, (q) “ACL injury” AND “adolescent” AND “BPTB”.

Titles and abstracts of studies identified were screened by two authors independently. If they met the eligibility criteria, they were evaluated for their full text. A search using ResearchGate was done in order to evaluate papers reported in sections “references” and “citations” of potentially relevant original articles found during the previous step. Finally, a search using “similar articles” was done for papers selected. Studies found were discussed by the authors before including them into the review.

## Results

### Study selection

**Fig. 2:**
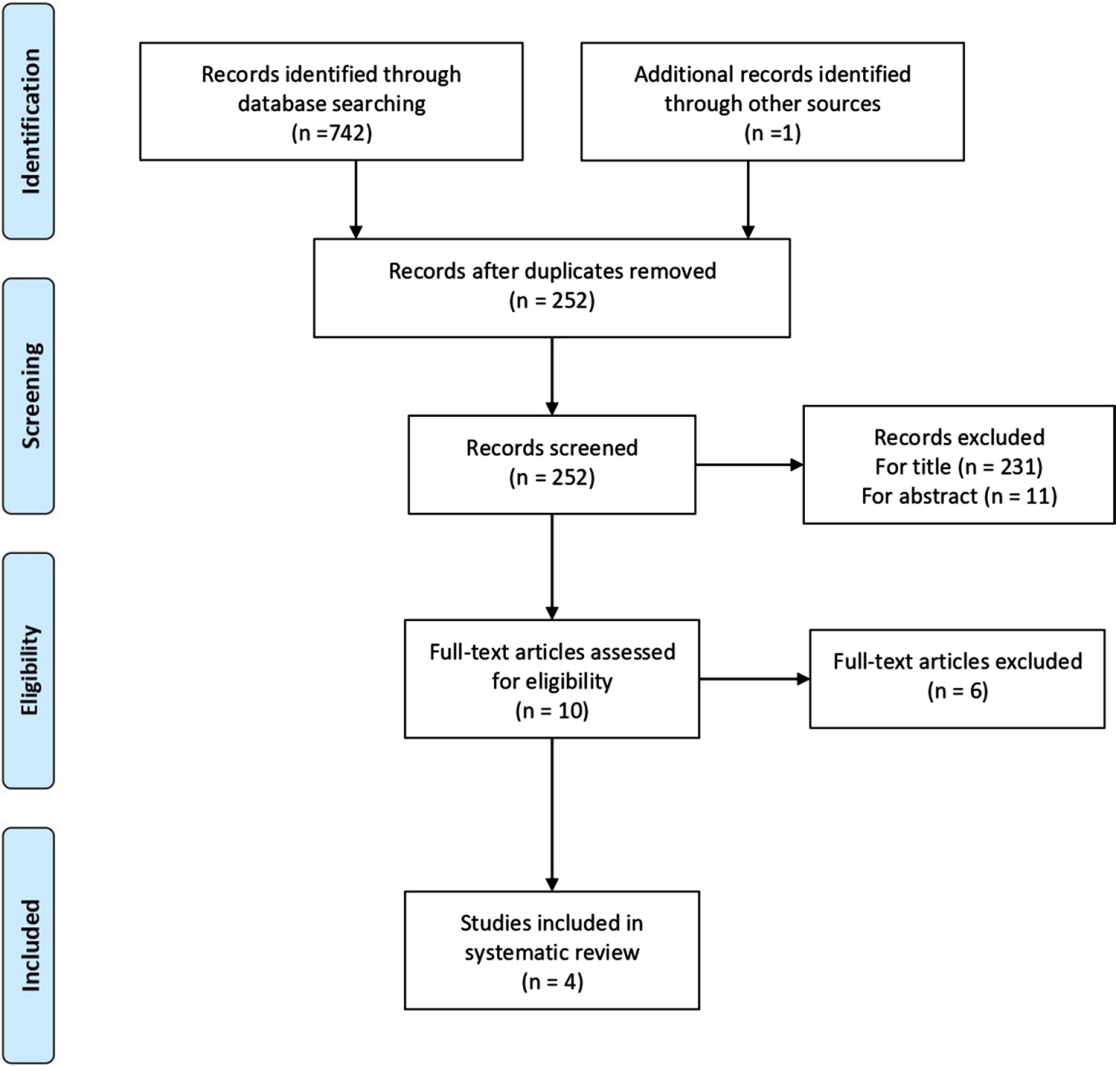
Prisma flow diagram

The search results are shown in Fig. 2, according to PRISMA guidelines. The search identified 742 results of which 738 did not satisfy the eligibility criteria and so they were excluded. Studies in which the reconstruction with patellar tendon was reinforced with extra-articular procedure, such as the one by McCarrol et al. [8], were excluded from the review too. In total four studies were considered and processed for data extraction.

### Methodological study quality assessment

The Newcastle–Ottawa scale [27] (NOS) was used to grade the methodological quality of each study assessed in this review (Table 1). In summary, the NOS scale uses a systematic approach based on 3 specific criteria: Selection (S), Comparability (C) and Exposure (E), which are subdivided in 9 criteria: (S1) representativeness of the exposed cohort; (S2) selection of the non-exposed cohort; (S3) ascertainment of exposure; (S4) demonstration that outcome of interest was not present at start of study; (C1) comparability of cohorts; (C2) controls on the basis of the analysis; (E1) assessment of outcome; (E2) follow-up long enough for outcomes to occur; (E3) Adequacy of follow-up of cohorts. Each study could have a maximum score of 9.

**Table 1:** Newcastle–Ottawa Scale

Study	Selection				Comparability		Exposure			Number of stars
	S1	S2	S3	S4	C1	C2	E1	E2	E3	
Memeo et al.	X	X	X		X	X	X		X	7
Shelbourne et al.	X	X	X	X	X	X	X	X	X	9
Fuchs et al.	X	X	X	X	X	X	X	X	X	9
McCarrol et al.	X	X	X		X	X	X	X	X	8

### Study characteristics

Main features of every study are summarized in Table 2. All the studies were cohort studies, only one of them was prospective [28], the others were retrospective [1, 28, 29]. The number of patients included in the studies ranged from ten to 60, with a total amount of 96 cases, of which 54 were males, while 42 were females. The mean age at surgery was 14.2 years (range 9–17) and the follow-up averaged 49.5 months (range 15–84). In three studies [28–30], the ACL was reconstructed with the BPTB autograft, while Fuchs et al. used the BPTB allograft [1].

**Table 2:** Characteristics of the studies included

Authors (year)	No. of patients (M/F)	Mean age (Range)	Maturity	Surgical technique	Months of follow-up (SD/Range)
Memeo et al. (2012)	10 (8/2)	14,4 (range 13–16)	All Tanner 3	Patellar tendon autograft	24.9 (range: 15–44)
Shelbourne et al. (2004)	16 (11/5)	14.8 (range 13–15)	7 Tanner 3	Patellar tendon autograft	Subjective test: 67.2 ± 26.4 (range: not reported)
			9 Tanner 4		Clinical test: 40.8 ± 13.2 (range: not reported)
Fuchs et al. (2002)	10 (6/4)	13.2 (range: 9–15)	All wide open tibial and femoral physes	Patellar tendon allograft	40 (range: 26–60)
McCarroll et al. (1994)	60 (29/31)	14.2 (range 13–17)	All closing physes	Patellar tendon autograft	50.4 (range 24–84)

### Main outcomes

The outcomes are summarized in Table 3. Studies reported 5 (5.2%) new ruptures out of 96 patients. Memeo et al. observed a re-injury associated with a meniscal medial tear after a traumatic knee sprain in a soccer player [28]. Shelbourne et al. described one ACL re-tear in a patient involved in a motor-cross accident three years after surgery, while two patients suffered of ACL tear in their contralateral knees, respectively, one and three years after surgery [30]. McCarroll et al. stated that three patients tore their ACL grafts and one patient had a subsequent meniscal tear. They also reported that two patients required arthroscopic ACL cyclops resections in order to treat lack of extension occurred after ACLR [29].

**Table 3:** summary of reviewed studies

Authors (year)	Clinical and instrumental evaluations	Score applied	Outcomes of study	Complications reported
Memeo et al. (2012)	Growth disturbances, early physeal arrest, limb discrepancy, angular deviation, KT 1000	Orthopadische Arbeitsgruppe Knie Score (OAK)	Return to sport rate: 100%, no alteration noticed in clinical and instrumental tests, OAK scored good to excellent except for one fair	Re-injury rate: 10%
Shelbourne et al. (2004)	Growth disturbances, early physeal arrest, limb discrepancy, angular deviation, medial or lateral joint space narrowing, knee range of motion, growth in height, isokinetic quadriceps muscle strength, KT 1000, Lachman test, mean growth since surgery	IKDC 2000 subjective score, Modified Noyes knee Questionnaire (MNKQ)	Return to sport rate: 100%, no alteration noticed in clinical and instrumental tests, mean growth since surgery was 8.7 cm (range 5.1;16.5), IKDC averaged 95.4 ± 6.9, Modified Noyes Knee questionnaire averaged 97.6 ± 2,9	Re-injury rate: 6.25%, contralateral ACL tear rate: 12.5%
Fuchs et al. (2002)	Early physeal arrest, limb discrepancy, angular deviation, range of motion, KT 1000, mean growth since surgery	IKDC 2000 subjective score, Lysholm-Gillquist score (LGS)	Return to sport rate: 90, no alteration noticed in clinical and instrumental tests, mean growth since surgery was 10 cm, IKDC scored 7 normal, 2 almost normal and 1 abnormal, LGS averaged 95 (9 excellent and 1 fair)	Re-injury rate: 0%
McCarrol et al. (1994)	Growth disturbances, limb discrepancy, angular deviation, KT 1000, mean growth since surgery	NA	No alteration noticed in clinical and instrumental tests, mean growth since surgery was 2.3 cm (range: 0;10.2)	Re-injury rate: 5%

Growth disturbances were assessed through different ways: Memeo et al. and Fuchs et al. performed clinical exams [1, 25], while Shelbourne et al. and McCarroll et al. [29, 30] used both clinical and radiological tests. Neither growth plate disturbances and arrests [29, 30], nor evidence of varus or valgus angulation and limb length discrepancy [1, 28–30] were reported. No one found any alteration of growth, the height increasing after surgery was reported by three studies [1, 29, 30] with a mean of 4.39 cm (range 0–16.5). Shelbourne et al. and Fuchs et al. didn’t observe any pathological reduction in the range of motion of the injured knee [1, 30]. Only Shelbourne et al. evaluated the medial and lateral joint space narrowing and the isokinetic quadriceps muscle strength without finding any alterations [30].

### Secondary outcomes

All the studies reported a high rate of return to sport ranging from 91.7 to 100% [1, 28–30]. Patients returned to their preinjury level of daily activity and athletic participation.

Different patient-reported outcome measures (PROMs) were used to evaluate knee conditions. Two studies adopted the IKDC subjective knee evaluation form [1, 30], Shelbourne et al. reported a mean score of 95.4 ± 6.9 [30], while Fuchs et al. reported the grading distribution with seven grade A (normal), two grade B (nearly normal) and only one grade C (abnormal) [1]. In association with the IKDC, Fuchs et al. reported a mean Lysholm knee score of 95 points of 100: 90% patients reported excellent results and one patient reported a fair result [1]. Shelbourne et al. used instead the Modified Noyes Knee Questionnaire with a mean score of 97.6 [30]. Finally, Memeo et al. with the Orthopadische Arbeitsgruppe Knie Score (OAK) got three excellent, six good and one fair results with an average score of 87.7 [28]. No paediatric PROMs were utilized.

KT-1000 was performed by every study [1, 28–30] in order to test knee instability after ACLR [31]. All the authors reported good results. Only five patients (5.2%) belonging to the studies of Memeo et al. and McCarroll et al. presented a KT-1000 side-to-side difference higher than 5 mm [28, 29].

## Discussion

Historically, the most important concerns about transphyseal techniques were related to physeal damages and growth disturbances [9, 32]. Generally, soft tissue grafts are recommended in skeletally immature patients, however BPTB graft are occasionally utilized also in young patients [33].

The main finding of this study is that transphyseal ACLR with BPTB in patients who have almost achieved skeletal maturity is a technique with reported good clinical outcomes with low graft rupture rate without growth disturbances in a selected population.

Indeed, in our review of all the studies, no evidence of growth disturbances, neither in clinical nor in radiological test were reported. In particular, authors highlighted the absence of lower limb discrepancy, early physeal arrest, varus or valgus angulation, medial or lateral joint space narrowing.

However, an MRI study concerning focal bone bridge after this technique was not already performed. The only physical alteration is a lack of extension occurring two patients reported by McCarroll et al. in 1994 [29]. More recent studies did not describe this complication. This statement may be linked to different factors. First of all, McCarrol et al. performed surgery using prevalently an open procedure by mini arthrotomy or through the patellar defect [29]. In fact, only three patients out of 60 underwent an arthroscopically assisted procedure. Secondary, more recent studies can rely on improvements in BPTB technique and better experience of the surgeons due to the increasing rate of ACLR in the skeletally immature patient. These aspects may have led to fewer complication rate of surgical procedure.

We believe that a meticulous growth potential evaluation is another essential point to plan the adequate surgical technique: chronological age, skeletal age, knee growth plates maturity evaluation and Tanner staging should be considered in the selection of the graft and of the surgical technique [13, 34, 35]. Fuchs et al. observed open femoral and tibial growth plates in all ten patients and reported preoperative sexual maturity and height [1]. At the time of surgery, Shelbourne et al. evaluated Tanner staging, growth plates in weightbearing X-rays and height. They described 16 patients with clearly open growth plates and Tanner stage 3 and 4 (seven and nine patients, respectively) [30]. Memeo et al. performed an evaluation of the maturity before the surgery through the assessment of Tanner staging, X-rays and MRI. All patients presented with Tanner stage 3 with radiological evidence of open tibial and femoral physes [28]. McCarrol et al. evaluated preoperatively Tanner staging, adolescent growth spur, height and X-rays to determine if the patients could be considered skeletally mature.

Another important feature which influences the choice of the graft is the failure rate. In our review the authors reported five graft failures out of 96 patients treated, it means 5.2%. This value suggests that patellar tendon graft is also less likely to fail compared to the overall rate of ACL graft failure in children and adolescent patients. According to Ho et al., in fact, graft failure was identified in 9.6% of the 561 patients who underwent ACLR [36]. Results achieved by Ho et al. reflect the ones found in our review. They reported similar incidence of patellar tendon graft failure, 6%, confirming that this type of graft has the lowest failure rate in this kind of population. Patients treated with soft tissue graft, in fact, had more than twice the probability (13%) to have a re-rupture. These findings are confirmed by different studies on hamstring graft including the ones by Cohen et al. [37], Calvo et al. [38] and Pennock et al. [39] which reported a failure rate of 11, 12 and 21, respectively.

All the authors reported good results in terms of anterior–posterior stability considering KT-1000 side-to-side difference [1, 28–30]. Studies included in our review reported 91 KT-1000 side-to-side difference lower than 5 mm and 5 higher [29]. Thus, 5.2% of ACLR using patellar tendon generate a certain degree of laxity. Anyway, this is not necessarily related to symptomatology, in fact, McCarroll et al. stated that no one of the three athletes complained episodes of giving way [29].

Wong et al. in their meta-analysis of ACL rupture in skeletally immature subjects reported the average result of IKDC and Lysholm score obtained from 23 and 20 studies, respectively [40]. The IKDC score ranged from 81 to 100 with 88% of grade A or B, the Lysholm score had a mean value of 94.6 points. In our review, we reported similar outcomes, however a prospective controlled group is recommended to better compare different techniques in skeletally immature patients.

Patellar tendon graft has been described by Memeo et al., Shelbourne et al. and Fuchs et al. as the correct choice for patients who do not want to modify their activity level after ACLR [1, 28, 30]. According to Kay et al. [41], the overall rate of return to preinjury level after ACLR is 78.6%, while in our review the return to sport using patellar tendon is 93.8%. Actually, the range of age evaluated in the meta-analysis is wider, considering children from 6-year-old, but the mean age is perfectly comparable with the one of the studies included in our review.

The review has some limitations. The first one concerns the sample, and it can be divided into two topics: quantity and quality. Quantity: the limited number of patients enrolled by the four studies included in the review, only 96, might not be large enough to represent the entire population. Quality: different choices of graft (allograft vs autograft) and different procedures (arthrotomy vs arthroscopy) were performed so it could dilute the methodology. However, considering that all the papers showed similar good results, independently from the graft or the procedure performed to reconstruct the ACL, we believe that these differences do not affect the safeness of BPTB technique. A second limitation is due to the lack of recent studies about the topic, in fact, papers analysed were published from 1994 to 2012, therefore the results achieved might be obsolete because of changing in different aspects such as: surgery, post-op rehabilitation, follow-up and prevention of re-injury.

Another limitation is due to the age of the patients enrolled. Since the mean age at surgery was 14.2 years it is difficult to establish the real occurrence of growth abnormalities. Having younger patient would grant better confidence for this objective. Unfortunately, the literature is lacking in studies about ACL reconstruction with BPTB technique in the youngest.

The last limitation is represented by the design of the studies included in the review, in fact there are no control groups, and all the data were collected in a retrospective way.

## Conclusion

Due to increasing involvement in highly competitive sport, the amount of ACLR in children and adolescents is getting higher. Surgery goal is to restore the preinjury activity level in the safest way possible with a low rate of ACL graft re-rupture. Patellar tendon graft could be a good choice in patients Tanner 3 and 4 with this aim. In fact, good clinical outcomes with a low graft rupture rate were reported without growth disturbances in this selected population. Further clinical and instrumental studies are recommended to better understand the real role of BPTB graft in a wider cohort of skeletally immature patients.

## Data Availability

Manuscript has not associated data in a data repository. If you need information please contact the corresponding author at the following address: marco.turati@unimib.it.
